# Deciphering the Global Proteomic Profile Involved in Methylmercury-Induced Cerebellar Neurodegeneration and Motor Dysfunction in Adult Rats

**DOI:** 10.3390/toxics10090531

**Published:** 2022-09-09

**Authors:** Leonardo Oliveira Bittencourt, Pedro Philipe Moreira Matta, Priscila Cunha Nascimento, Luciana Eiró-Quirino, Walessa Alana Bragança Aragão, Aline Dionizio, Luanna Melo Pereira Fernandes, Márcia Cristina Freitas Silva, Marília Afonso Rabelo Buzalaf, Michael Aschner, Maria Elena Crespo-Lopez, Cristiane Socorro Ferraz Maia, Rafael Rodrigues Lima

**Affiliations:** 1 Laboratory of Functional and Structural Biology, Institute of Biological Sciences, Federal University of Pará, Belém 66075-110, PA, Brazil; 2 Department of Biological Sciences, Bauru School of Dentistry, University of São Paulo, Bauru 17012-90, SP, Brazil; 3 Department of Morphology and Physiological Sciences, Center of Biological and Health Sciences, State Unversity of Pará, Belém 66087-662, PA, Brazil; 4 Department of Molecular Pharmacology, Albert Einstein College of Medicine, Bronx, NY 10461, USA; 5 Laboratory of Molecular Pharmacology, Institute of Biological Sciences, Federal University of Pará, Belém 66075-110, PA, Brazil; 6 Laboratory Pharmacology of Inflammation and Behavior, Institute of Health Sciences, Federal University of Pará, Belém 66075-110, PA, Brazil

**Keywords:** proteomic, neurotoxicology, neurodegeneration, organic mercury, microglia, oxidative stress

## Abstract

Mercury is a ubiquitous pollutant in the environment with potential neurotoxic effects. Several populations are susceptible to mercurial exposure, especially methylmercury (MeHg) at low doses for long periods through food consumption. Given this, the present work aimed to assess the effects of long-term MeHg exposure on the cerebellum of rats from a translational perspective using a representative dose, assessing molecular, biochemical, morphological, and behavioral parameters. The model was produced by administering 40 µg/kg of MeHg for 60 days to adult male Wistar rats by oral gavage. As a result of this exposure, the animals presented motor deficits in open field and rotarod tests which were associated with an increase in total mercury content in cerebellar parenchyma, a reduction in antioxidant competence against peroxyl radicals, and increased nitrite and lipid peroxidation levels. The proteomic approach showed 317 modulated proteins. Such findings were associated with reductions in mature neuron and Purkinje cell densities and glial fibrillary acidic protein immunostained areas and increased microglial density. In addition, decreases in myelin basic protein and synaptophysin immunostaining were also observed. The results thus provided new evidence of the mechanisms underlying complex MeHg-induced neurodegeneration, especially the proteins underlying the biochemical and morphological features associated with motor dysfunction.

## 1. Introduction

According to the World Health Organization, mercury (Hg) is one of the most toxic compounds, and it has been characterized as a public health threat [1]. It enters the environment from volcanic eruptions and atmospheric cycling, along with anthropogenic activities, such as industrial processes, and through illegal mining that leads to contamination not only of the water table but also of the workers in these mines through the vapor that is emitted during the gold purification process [2,3,4].

Through methylation promoted by sulfate-reducing bacteria, especially in aquatic ecosystems, inorganic Hg is transformed into methylmercury (MeHg), a highly toxic organic compound that biomagnifies and bioaccumulates in aquatic environments; therefore, the ingestion of seafood from contaminated areas is considered the main route of chronic human exposure [2,4].

One of the main targets of MeHg is the central nervous system (CNS), where it exerts neurological effects, including visual disturbances, ataxia, paresthesia, neurasthenia, hearing loss, and neurodegeneration, among others [5]. However, it is important to state that the clinical features are heterogeneous, depending on the age of the individual exposed, the length of exposure, and the amount of mercury ingested, among other factors. Various agencies, such as the World Health Organization and the US Environment Protection Agency (USEPA), have recommended maximum weekly consumptions of 1.6 and 0.7 µg/Kg of MeHg, respectively, equivalent to approximately 0.046 and 0.02 µg/g in the brain [4]. However, these are “provisional” recommendations that depend on new evidence to be re-evaluated. For example, Indigenous peoples and other riverine communities in the Amazon are continuously exposed to MeHg in food due to small-scale illegal gold mining activities, rainforest deforestation, soil erosion, and hydroelectric plant construction [4,6]. Thus, the unfortunate realities still faced by populations vulnerable to mercurial exposure caused by illegal anthropogenic actions and where there is no proper risk- and damage-management planning or government assistance are reinforced.

Our research group has investigated the harmful effects of chronic exposure to mercury in recent years. The choice of dose was based on a protocol described elsewhere [7] that was adapted by our group [8]. Using this previous protocol, we found changes in the hippocampal region [9], the motor cortex [10], and the spinal cord [11]. The data have shown that MeHg can trigger motor and cognitive deficits associated with oxidative stress at this dose and in this exposure window as well as neural and glial cell damage and modulation of proteomic profiles consistent with neurodegenerative process.

From this perspective, motor functions are a set of abilities supported by the consonance between the peripheral and central nervous systems (CNS). Thus, in the CNS, the motor cortex, basal nuclei, spinal cord, and cerebellum are intrinsically associated with the planning, execution, and modulation of such abilities [12]. The cerebellum is mainly responsible for integrating sensorimotor information from the brain and spinal cord and plays a pivotal role in balance, coordination, and motor refinement. Considering the fundamental importance of this organ and the neurological outcomes of MeHg intoxication, most of the motor signs and symptoms caused by MeHg, such as ataxia and tremors, are mediated by cerebellar dysfunction. Therefore, considering the fundamental importance of the cerebellum to motor abilities and the clinical outcomes observed in people exposed to MeHg, what would be the cerebellar proteomic profile underlying the biochemical and morphofunctional impairments?

Accordingly, this study aimed to investigate the effects of MeHg exposure on the cerebellums of adult rats in a low-dose, long-term model. In order to examine possible cerebellar changes in rats, we carried out behavioral, oxidative biochemistry, morphological, and immunohistochemistry analyses of the rat cerebellums.

## 2. Materials and Methods

### 2.1. Experimental Animals and Ethical Aspects

In this study, 54 male albino rats (*Rattus norvegicus*, Wistar strain) with body weights between 200 g and 250 g (90 days old) were obtained from the Central Animal Facility of the Institute of Biological Sciences from the Federal University of Pará, with the approval of the Ethics Committee on Animal Use (CEUA) under the protocol number 225-14. The animals were housed in polypropylene cages, divided into groups of up to 4 animals per cage. The animals received water and food ad libitum and were kept at a temperature of 25 °C with a 12 h dark/light cycle (lights on at 7 a.m.). In agreement with our previous studies that investigated the molecular features underlying MeHg toxicity on different organs in rats, we only used male rats in this experimental design in order to avoid possible biases triggered by neuroendocrine factors in female rats, such as the estrous cycle, that would affect not only the comparability between the groups but would also lead to discrepancies in the cerebellar proteomes of the control and exposed animals.

**Figure 1:**
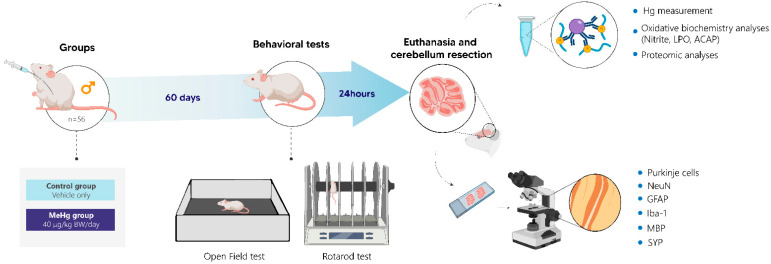
Methodological summary of the study. Wistar rats were divided into control (n = 27) and MeHg groups (dose of 40 µg/kg/day) (n = 27). After 60 days of MeHg exposure, the motor functions were assessed by open field and rotarod tests. Then, after cerebellum collection, the samples were processed for total mercury determination, oxidative biochemistry (antioxidant capacity against peroxyl radicals (ACAP), nitrites, and lipid peroxidation (LPO)), and proteomic and morphological analyses (Purkinje cell count, immunohistochemistry for mature neurons (NeuNs), glial fibrillary acidic protein (GFAP), microglial markers (Iba1), myelin basic protein (MBP), and synaptophysin (SYP)).

The animals were divided into two different groups: (1) the exposed group (27 animals), which received a daily dose of 40 µg/kg of MeHg (MeHg chloride; Sigma-Aldrich, St. Louis, MO, USA) solubilized in corn oil and administered by intragastric gavage; (2) and the control group (27 animals), which received only the vehicle daily. In this way, the dose used in this work simulated daily human consumption levels in endemic regions of mercurial exposure [7,8,10,11,13]. After 60 days of exposure, the animals were submitted to behavioral tests, then they were anesthetized and euthanized for cerebellar collection or perfusion for morphological assessment. The methodological steps are displayed in Figure 1.

### 2.2. Behavioral Tests

The tests were conducted in a sound-attenuated room under low-intensity light (12 lux), with habituation for at least one hour before the assays were begun.

The open field test evaluated the spontaneous locomotion of the animals in an acrylic arena (100 × 100 × 40 cm) with a floor divided into 25 equal quadrants (20 × 20 cm) and a digital camera positioned on the ceiling. Each animal was placed in the center of the arena, filmed, and observed for 5 min. The parameter of total distance traveled was analyzed by ANY-maze software (Stoelting Co., Chicago, IL, USA), while the number of standing positions (rearings) was measured manually.

The rotarod test evaluated the animals’ forced motor coordination. Initially, they were submitted to a training session on the axis of rotation of the equipment for 120 s at 15 revolutions per minute (RPM). After training, the animals were submitted to subsequent sessions with increasing rotations for 120 s each (16, 20, 25, 28, and 37 RPM). The number of falls was recorded for each session.

### 2.3. Sample Collection

After the behavioral tests, a set of animals were deeply anesthetized with a mixture of ketamine hydrochloride (90 mg/kg) and xylazine hydrochloride (9 mg/kg) and euthanized by exsanguination. The brains were removed from the skull to collect the cerebellums, and the samples were gently washed in cold saline buffer, divided into two hemispheres, frozen in liquid nitrogen, and stored at −80 °C until the performance of further analyses.

### 2.4. Total Mercury Content Measurement

The total mercury contents in the samples were determined by cold vapor atomic absorption spectrometry (CVAAS) using a semi-automatic mercury analyzer (model Hg-201, Sanso Seisakusho Co. Ltd., Tokyo, Japan), as established by Suzuki et al. [14] and previously reproduced [9]. A total of 0.5 g of wet sample was digested in an acid solution of nitric acid, perchloric acid, and sulfuric acid (1 : 1 : 5, *v*/*v*; Sigma-Aldrich, USA) on a hot electric plate (210 °C) for 30 min. The basis of this method is the conversion of Hg^2+^ into Hg^0^ after the addition of stannous chloride (Sigma-Aldrich, USA), in which the vapor generated is conducted by the duct system and automatically guided by a 4-way valve to an absorption cell after refrigeration. The samples were inserted in the absorption cell, in which the absorbance was measured at 253.7 nm to estimate the levels of the metal after comparison with a standard curve. The results were expressed as μg/g. 

### 2.5. Oxidative Biochemistry Assays

For these analyses, the samples were thawed and resuspended in 20 mM Tris-HCl buffer (pH 7.4, at 4 °C) for sonic disintegration (~1 g/mL). Homogenization was performed with the help of an ice bath below the microtubes to avoid any type of heat that could affect the lipids and proteins in the samples. All biochemical tests were carried out in triplicate and used the supernatants after centrifugation. 

The analysis of antioxidant capacity against peroxyl radicals (ACAP) was determined as previously described by Amado et al. [15]. The supernatant of each sample was obtained after centrifugation (10,000× *g*, 20 min at 4 °C) and exposed to a peroxyl radical generator (2,2′-azobis 2 methylpropionamidine dihydrochloride (ABAP); 4 mM) or ultrapure water, in triplicate. After the reaction, the fluorescence was measured in a fluorimeter (Victor X3, Perkin Elmer, Waltham, MA, USA) at 35 °C. After the first reading to determine background fluorescence values, a total of 10 μL of 2′,7′ H_2_DCF-DA (40 nM) was added to the microplate and read every 5 min for 60 min. The relative difference between reactive oxygen species areas with and without ABAP was considered as a measure of antioxidant capacity. The results were transformed to the inverse of the relative areas and expressed as percentages of the control group.

In the nitrite level assessment, an aliquot of the crude homogenate was centrifuged at 14,000 rpm for 10 min at 4 °C to collect the supernatant. The protocol used was established by Green et al. [16] and consisted of incubating 50 µL of a sample or standard (sodium nitrite) with Griess’ reagent (naftil-ethylene-diamine 0.1% and sulfanilamide 1% in 5% phosphoric acid (1:1)) at room temperature. The absorbance was measured at 550 nm and compared with the standard curve of sodium nitrite. All analyses were performed in triplicate, and the data were normalized by total protein content based on Bradford’s method [17]. The values were expressed as µmol/mg of protein and converted to percentages of the control group.

### 2.6. Proteomic and Bioinformatic Analyses

These experiments were conducted in biological triplicates, using 3 samples per group, according to the process described below. Originally, 6 animals from each group were used, and two different cerebellums from the same group were pooled into a single sample. The samples were cryofractured in liquid nitrogen using a cryogenic mill, with subsequent extraction of soluble proteins with lysis buffer (7 M urea, 2 M thiourea, 40 mM dithiothreitol—all diluted in ammonium bicarbonate solution) under constant stirring at 4 °C. Afterwards, the samples were centrifuged for 30 min at 14,000 rpm at 4 °C to collect the supernatant and determine the amount of protein by Bradford’s method [17]. Subsequently, a fixed amount of protein (50 μg) from each sample was supplemented with ammonium bicarbonate (50 mM) to a volume of 50 μL in order to reach a final concentration of 1 μg/μL. For each sample, 10 μL of 50 mM ammonium bicarbonate and 25 μL of 0.2% RapiGEST™ (Waters Co., Manchester, UK) were added, followed by incubation at 37 °C for 30 min. Then, 2.5 μL of 100 mM dithiothreitol was added, followed by incubation at 37 °C for 60 min, after which 2.5 μL of 300 mM iodoacetamide was added prior to incubation for 30 min at room temperature (in the dark). Subsequently, 10 μL of trypsin was added and enzymatic digestion occurred for 14 h at 37 °C, with subsequent addition of 10 µL of 5% trifluoroacetic acid and incubation for 90 min at 37 °C and posterior centrifugation at 14,000 rpm at 6 °C for 30 min. Afterwards, the supernatant was collected and purified/desalted using C18 Spin columns (Pierce, cat. no. 898705). After digestion, 10 µL of 5% trifluoroacetic acid was added and vortexed and incubated at 37 °C for 90 min. Next, centrifugation was performed at 14,000 rpm at 6 °C for 30 min and the supernatant was transferred to individual vials (Waters Total Recovery Vial, Waters) and quantities of 5 μL of 1 pmol/μL of alcohol dehydrogenase (MassPREP Digestion Standard Alcohol Dehydrogenase, cat. no. 186002328, Waters) were added to the samples, which were then vortexed. A total of 85 μL of 0.1% formic acid in 3% acetonitrile was added. After the preparation of the samples, they were submitted to Nano-UPLC (Nano-Ultra Performance Liquid Chromatography; Waters), a liquid chromatography process that allows high-performance protein separation, for later identification by mass spectrometry. The identification of the peptides was performed in a nanoACQUITY UPLC-Xevo QTof MS system (Waters, Manchester, UK). The nanoACQUITY UPLC^®^ is equipped with a nanoACQUITY HSS T3 reversed-phase analytical column (75 μm × 150 mm, 1.8 μm particle size; Waters, Manchester, UK). The column was equilibrated with 93% mobile phase A (0.1% formic acid in water) and 7% mobile phase B (100% acetonitrile + 0.1% formic acid). Then, the peptides were separated with a linear gradient of the mobile phase from 7–85% (0.1% formic acid in 100% acetonitrile) for 70 min at a flow rate of 0.35 μL/min. The column temperature was kept at 35 °C. 

The Xevo^®^ G2 Q-TOF mass spectrometer was operated in positive ionic nanoelectrospray mode, and data were collected using the MSE method at high energy (19–45 V), which allows the acquisition of data for both precursor ions and fragments in a single injection. The source conditions used included: 2.5 kV capillary voltage, 30 V sample cone, 5.0 V extraction cone, and 80 °C source temperature. Data were acquired over 70 min, with a sweep in the range of 50–2000 Da. The lockspray, which was used to ensure accuracy and reproducibility, was operated with a solution of [Glu1] fibrinopeptide (1 pmol/μL), with a flow of 1 μL/min, as a reference ion in positive mode at m/z 785.8427. The identification of the peptides was performed via UPLC-Xevo QTof MS (Waters, Manchester, UK), using the Protein Lynx Global Server (PLGS). The protein identification was performed by downloading the Uniprot database (entry number UP000002494, downloaded in January 2022) and was deposited in the PeptideAtlas repository under number PASS02779. Then, bioinformatical analyses were executed in Cytoscape (v. 3.6.1, Java^®^) with the ClueGO Plugin for the determination of groups of biological processes based on the Gene Ontology (GO) [18] and ClusterMarker plugin to create a protein–protein interaction (PPI) network. 

For the over-representation analysis (ORA), proteins that were differentially expressed and unique were used. The data regarding the ratios of the previous analysis were processed, and cut values were applied for the screening of proteins with expression values of 50% above or below in the exposed group compared to the control. The analysis considered only proteins with log2ratio values ≤−0.58 or ≥0.58. Using the UNIPROT conversion tool (https://www.uniprot.org/uploadlists/, accessed on 1 July 2022), the protein codes were converted into Entrez gene IDs. For proteins with absolute changes, values of −1 (for proteins detected only in the control samples) and 1 (for proteins detected only in the exposed samples) were assigned. The R studio program was used with the EGSEA plugin [19,20]. In this step, the Uniprot database, made available by Bader Lab, was consulted to identify proteins and the biological processes they participate in. After these verifications, we used Cytoscape software [21] with the Enrichment Lab plugin pipeline to group the sets of proteins previously consulted, and after that the main biological processes were selected for graphical analysis.

Subsequently, a PPI analysis was performed (https://www.networkanalyst.ca/, accessed on 1 July 2022) [22] to construct a representative image according to the number of interactions between the proteins and the other proteins found to be altered, of which there were 50 proteins in total. A Circos plot was generated by the R studio program with the GOplot plugin.

### 2.7. Morphological Assessment

Another set of animals (n = 10 per group) were anesthetized with a mixture of ketamine hydrochloride (90 mg/kg) and xylazine hydrochloride (9 mg/kg) and perfused through the left ventricle with 0.9% heparinized saline followed by 4% paraformaldehyde. Then, the cerebellums were processed by a routine histological procedure, embedded in Paraplast (McCormick™), and sectioned at 5 µm using a microtome. Some sections were stained with hematoxylin and eosin (HE) to perform Purkinje cell number counts; other sections were selected for indirect immunohistochemistry with the following antibodies: Anti-NeuN (1:100; Chemicon, Darmstadt, Germany) [23,24]; Anti-GFAP (1:2000; Dako, Copenhagen, Denmark) [23,25]; Anti-Iba1 (1:1000; WAKO, Osaka, Japan) [26], Anti-MBP (1:100; Chemicon, Darmstadt, Germany) [26], and Anti-Synaptofysin (1:300; Millipore, Darmstadt, Germany) [27].

After revealing with diaminobenzidine (DAB), the slides for cell counting (anti-NeuN and anti-Iba1) were analyzed using a bright-field microscope at 40× magnification with a 0.0665 mm^2^ grid attached to the eyepiece which was also used for the Purkinje cell counts. Three sections per animal (from the exposed and control groups) and three fields per section were considered. After counting, photomicrographs were taken of the most representative fields for each group for each technique, using an Axioscope microscope equipped with a CCD AxioCam HRC color camera. 

To analyze the slides for the anti-GFAP, anti-MBP, and anti-synaptophysin immunohistochemistry analyses, photomicrographs were first taken at 40 × magnification (three sections per sample and three fields per section) and analyzed in ImageJ software. The photomicrographs were submitted to the color deconvolution plugin. Afterwards, the relative area fractions of DAB staining in the sections were determined [26,27,28].

### 2.8. Statistical Analyses

The data were analyzed using GraphPad Prism 7.0, and the data distribution was tested by the Shapiro–Wilk method. The Student’s *t*-test was used to analyze the open field behavioral test, total mercury determination, oxidative biochemistry assays, and cell counts (Neu-N, Iba-1, Purkinje cells). The rotarod test results were analyzed using a two-factor ANOVA test with Sidak’s post hoc test. For the analyses of area fractions (MBP, GFAP, and synaptophysin), the Mann–Whitney test was used, considering a significance value of *p* < 0.05. The proteomic results were analyzed using ProteinLynx Global SERVER™ (PLGS) software and the Monte Carlo algorithm, and proteins were considered under-regulated with *p* < 0.05 and over-regulated with 1—*p* < 0.95.

## 3. Results

### 3.1. Prolonged Exposure to MeHg Promoted Increased Mercury Levels in the Cerebellums of Wistar Rats

Total Hg levels in the cerebellums of adult rats were significantly higher after prolonged exposure to MeHg at 0.04 mg.kg^−1^ of body mass. The total Hg levels in the exposed group were 0.068 µg/g (±0.005; *p* < 0.0001) when compared to the control group (0.005 ± 0.003 µg/g; *p* < 0.0001).

### 3.2. Exposure to MeHg Promoted Modulation of Oxidative Biochemistry in the Cerebellums of Wistar Rats by Reducing Antioxidant Response and Increasing Pro-Oxidant Factors

**Figure 2:**
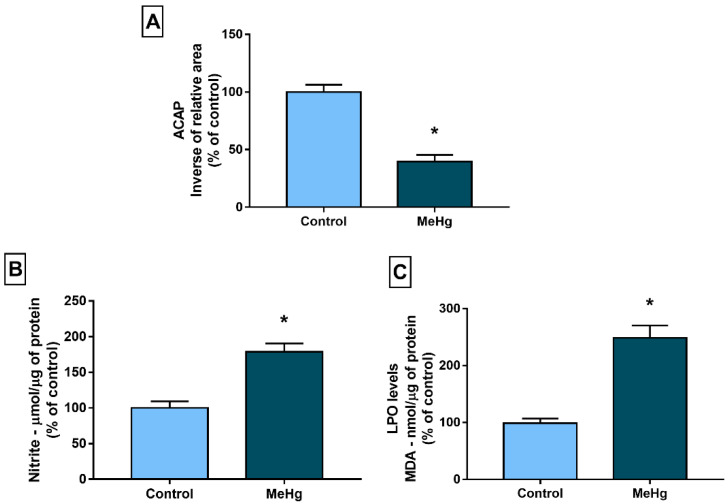
Effects of prolonged exposure to MeHg (40 µg/kg/day), for 60 days, on the oxidative biochemistry of the cerebellums of Wistar rats. (**A**) Antioxidant Capacity Against Peroxyl Radicals (ACAP). (**B**) Nitrite levels. (**C**) Lipid peroxidation (LPO) levels measured by malondialdehyde (MDA) levels. Results are expressed as means ± standard errors of the mean (SEMs) and converted to percentages of the control group. n = 10 per group. * *p* < 0.05. Student’s *t*-test.

A significant reduction was found in ACAP in the group exposed to MeHg (*p* < 0.0001; Figure 2A). On the other hand, exposure to MeHg promoted an increase in nitrite (*p* = 0.0001; Figure 2B) and LPO levels (*p* < 0.0001; Figure 2C).

### 3.3. Exposure to MeHg Promoted Changes in the Proteomic Profiles of the Cerebellums of Wistar Rats

**Figure 3:**
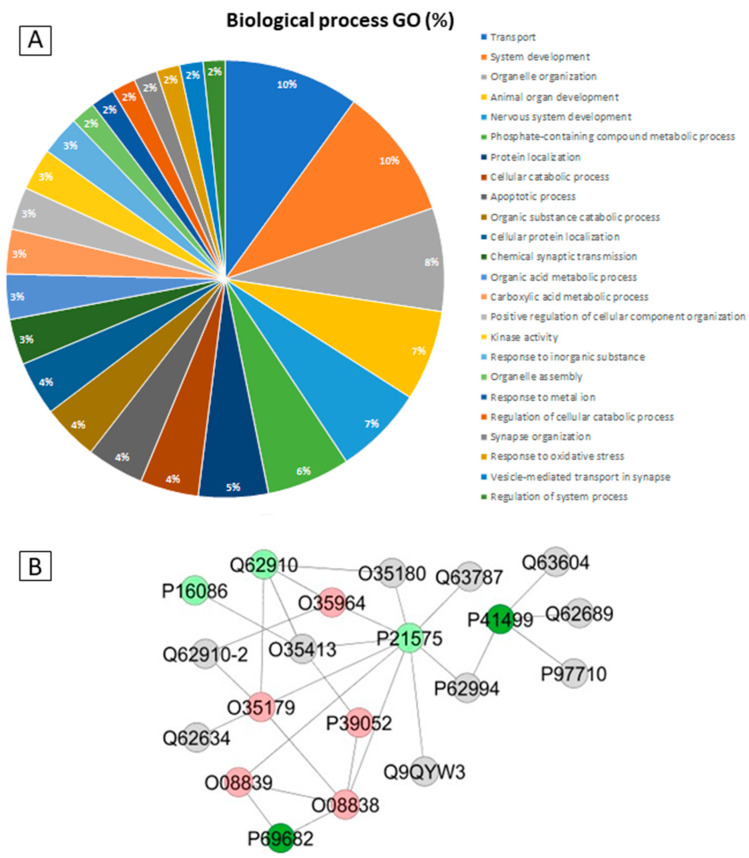
Functional distribution of proteins identified with differential expression in the cerebellums of adult rats between the MeHg group and the control group (**A**). Protein categories are based on the Gene Ontology (GO) of biological processes. (**B**) Protein–protein interaction network. Red and dark green colors indicate proteins found in the control and exposed groups, respectively; light green and pink colors indicate upregulated and downregulated proteins, respectively; gray nodes represent those proteins that were not identified in this study but which entered into interactions in the network.

Proteomic analysis revealed 317 proteins that were differentially regulated by MeHg, 244 of which were upregulated and 72 downregulated. In addition, 163 proteins were unique to the control group and 106 to the exposed group (Supplementary Table S1). The GO analysis pointed to 24 biological processes, of which the top 5 most altered were: transport (10%), system development (10%), organelle organization (8%), animal organ development (7%), and nervous system development (7%) (Figure 3A). The protein interaction network depicted a central node of a microtubule protein related to vesicular transport (P21575: Dynamin-1) which showed interactions with excitatory synapse regulatory protein (O08838: Amphiphysin) and another microtubule protein (P39052: Dynamin-2) (Figure 3B).

**Figure 4:**
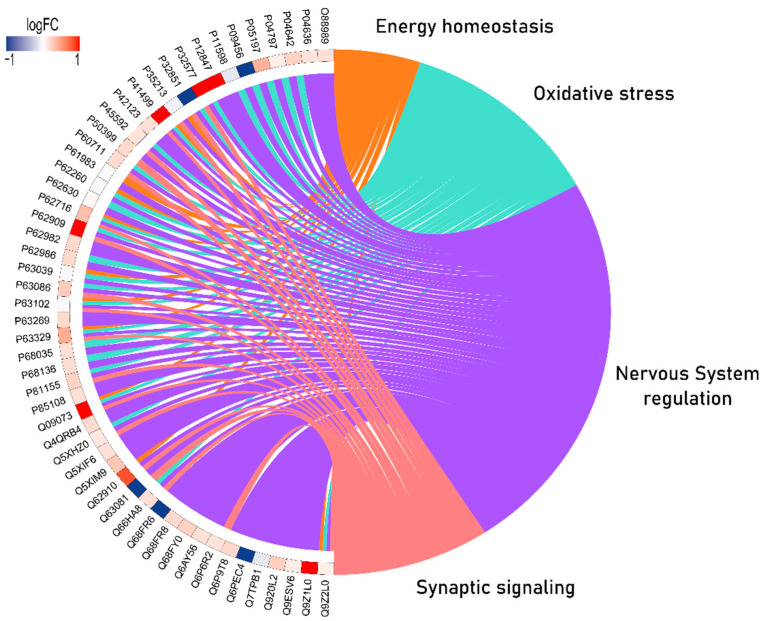
Circos plot of protein-protein interactions (PPIs) resulting from the over-representation analysis of the cerebellar proteomes of rats exposed to MeHg. The results are organized into the categories of energy homeostasis (orange), oxidative stress (light green), nervous system regulation (purple), and synaptic signaling (warm pink). Blue-scale proteins were downregulated and red proteins were upregulated in the MeHg group compared to the control group.

ORA analysis identified 52 proteins with greater PPI interactions with different regulatory statuses in response to MeHg treatment, which are associated with energy homeostasis, oxidative stress, nervous system regulation, and synaptic signaling (Figure 4).

### 3.4. Exposure to MeHg Promoted Morphological Changes in the Cerebellums of Wistar Rats

**Figure 5:**
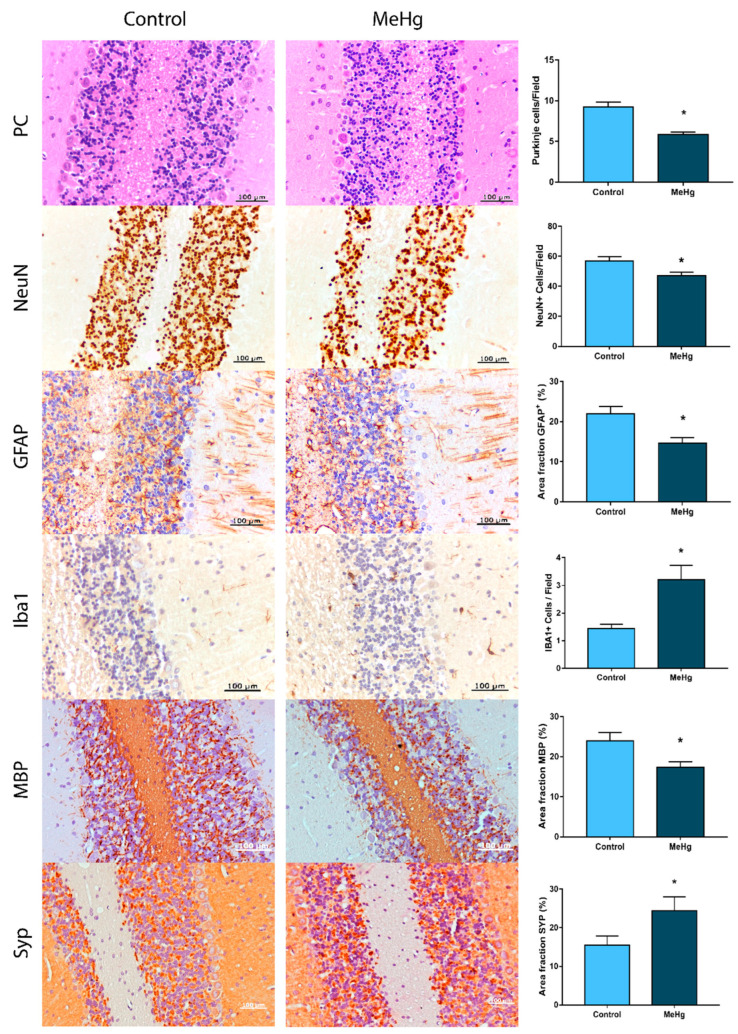
Effects of exposure to MeHg on the morphological integrity of cerebellums of Wistar rats. PC: Purkinje cells; NeuN: mature neurons; GFAP: glial fibrillary acidic protein; Iba1: microglia markers; MBP: myelin basic protein; Syp: synaptophysin. Results are expressed as means ± standard errors of the means (SEMs). The Student’s *t*-test was used to analyze the PC, NeuN, and Iba1 results, and the Mann–Whitney test was used to analyze the area fraction data. * *p* < 0.05.

Morphological analyses by HE staining (Figure 5) showed significant reductions in the densities of Purkinje neurons (*p* < 0.0001) and reductions in populations of NeuN+ cells (*p* = 0.0142). Moreover, exposure to MeHg reduced the area fractions immunostained with anti-GFAP in the rat cerebellums, followed by increased microglial cell density in molecular and granular layers (*p* < 0.05). Increased area fractions immunostained with anti-synaptophysin were noted in MeHg-exposed animals (*p* = 0.0350), concomitant with decreases in anti-myelin-basic-protein-immunolabeled area fractions (*p* = 0.0046). 

### 3.5. Exposure to MeHg Promoted Changes in the Spontaneous Motor Locomotion, Coordination, and Balance of the Rats

**Figure 6:**
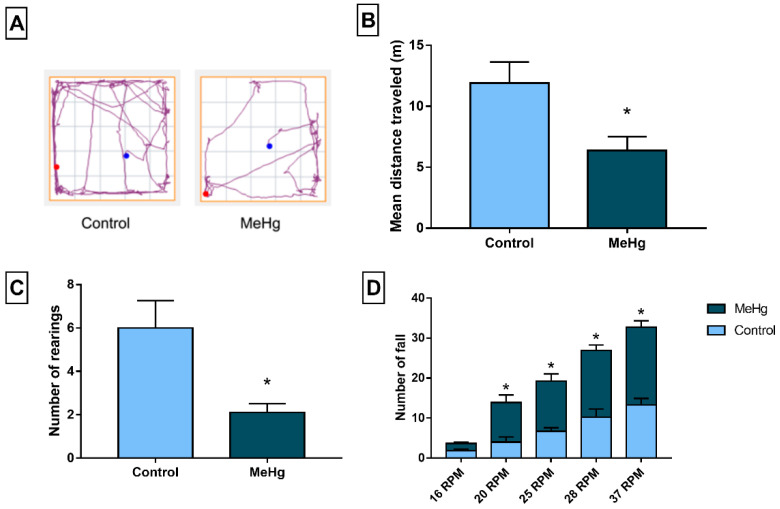
Effects of long-term exposure to MeHg (40 µg/kg/day) for 60 days on spontaneous and forced motor locomotion in Wistar rats. (**A**) Tracking plot of horizontal locomotor activity, where blue dots are for the initial position and red dots, final position (**B**) mean total distance traveled, and (**C**) number of rearings in the open field test. (**D**) Number of falls in the rotarod test. Results are expressed as means ± standard errors of the means (SEMs). n = 10 per group. The open-field parameters were analyzed using the Student’s *t*-test and the number of falls in the rotarod test by two-way ANOVA and Sidak’s post hoc test. * *p* < 0.05.

In the open field test, the MeHg group showed a reduction in locomotor activity (Figure 6A) characterized by reductions in horizontal exploration (*p* = 0.014; Figure 6B) and number of rearings (*p* = 0.008; Figure 6C) in comparison to the control group. In addition, the rotarod test showed changes in the balance and coordination of animals exposed to MeHg by an increase in the number of falls observed at 20 RPM, 25 RPM, 28 RPM, and 37 RPM compared to the control group (Figure 6D; *p* < 0.05).

## 4. Discussion

This novel study presents proteomic screening results related to multiple aspects of MeHg toxicity in rat cerebellums at doses representative of human consumption in endemic regions of mercurial exposure. The findings evidenced that MeHg-induced motor dysfunction was associated with a neurodegenerative pattern in the cerebellums of rats, namely, a reduction in the density of neurons and glial cells, followed by oxidative stress and global proteomic profile modulation. This is the first study that has revealed the cerebellar proteome underlying biological aspects from biochemistry to motor behavior after MeHg exposure, indicating an interesting regulation of proteins present in neurodegenerative diseases and essential biological processes, such as proteostasis, synaptic communication, and cell metabolism.

As previously mentioned, several regulatory organs have stated MeHg PTWIs, but there are considerable discrepancies between these safety guidelines and the realities faced by vulnerable populations. In this perspective, Crespo-López et al. [4] reviewed that MeHg content in fish meat, the main protein source for Amazonian riverine populations, can reach approximately 272 µg per week, almost three times the WHO’s recommendation. Although this datum is based on a conservative estimation due to the mathematical resources and data available in the literature, it highlights the criticality of this socioenvironmental issue. 

Our experimental protocol for MeHg exposure resulted in a total mercury mean concentration of 0.068 µg/g in the cerebellums of rats, and, in addition to the classical mercury neurotoxic effects, such as oxidative stress triggering, increase in mercury levels was associated with significant modulation of global proteomic profiles, affecting morphological components, such as myelin and synaptic communication, and resulting in glial damage and a neurodegenerative pattern consistent with cerebellum-related motor dysfunction. The mechanisms underlying mercury toxicity comprise molecular and biochemical factors, such as increased reactive oxygen species (ROS) production, and direct damage to DNA and microtubule polymerization [29], combined with excitotoxicity mediated by glutamate [30,31]. Our data point out that even at doses below the international recommendations, over long periods, MeHg exposure triggered oxidative stress in the rat cerebellums by decreasing antioxidant capacity against peroxyl radicals and increasing nitrite levels, leading to LPO. These three parameters come to be intrinsically associated once peroxyl radicals induce the oxidation of polyunsaturated fatty acids and the propagation of LPO, and several reactive nitrogen species can also impair unsaturated lipids, in addition to causing post-translational modifications (PTMs) in proteins, leading to S-nitrosylation [32,33,34,35].

Although several PTMs (phosphorylation, acetylation, methylation, e.g.,) naturally occur in cells in normal conditions, damage caused by genetic and epigenetic modulation and oxidative stress can affect cell functioning [36,37]. As reviewed by Ke et al. [38], several in vivo studies have pointed out that MeHg causes PTM damage, affecting kinase and phosphorylase activity and the ubiquitin system. Corroborating this, the ubiquitin–proteasomal system (UPS) also acts to prevent protein aggregation, which can lead to cell failure [39], and the proteomic approach revealed the downregulation of Proteasome subunit beta type-1 (P18421) and Ubiquitin carboxyl-terminal hydrolase isozyme L1 (Q00981) and the lack of expression of Proteasome subunit alpha type-1 (P18420) and Ubiquitin-conjugating enzyme E2 variant 2 (Q7M767). 

Another set of proteins related to proteostasis modulated by MeHg in the cerebellum are Heat Shock Proteins (HSPs). Considering their role as molecular chaperones involved in proteostasis through the maturation, re-folding, and degradation of proteins [40], HSPs have already been noted as therapeutic tools for various diseases, including neurodegenerative processes [41]. We found the upregulation of Heat shock protein 105 kDa (Q66HA8), Heat shock protein 75 kDa, mitochondrial (Q5XHZ0), Heat shock-related 70 kDa protein 2 (P14659), and Heat shock protein HSP 90-alpha (P82995), while Hsc70-interacting protein (P50503) and Hsp90 co-chaperone Cdc37 (Q63692) were absent in the MeHg-exposed group. In recent articles, our group has shown that HSPs are significant targets of organic and inorganic mercury exposure in animal models [8,9,11,42,43]. Thus, these proteins may be considered as potential predictors or biomarkers for neurological outcomes associated with mercurial exposure, mainly because the malfunctioning of both the HSP and UPS systems can lead to protein aggregation, which characterizes various neurodegenerative diseases, such as Alzheimer’s (AD), Parkinson’s, and Huntington’s diseases [44].

A previous study [9] reported the upregulation of apolipoprotein-E (ApoE) in the hippocampi of MeHg-exposed animals with memory impairments, which is often associated with AD and has also been investigated in populations exposed to mercury [45,46,47]. ApoE upregulation could represent a protective response against MeHg, since recent evidence has revealed the worsening of cardiovascular outcomes caused by MeHg in ApoE-knockout mice [48,49]. Moreover, we found overexpression of S100-B protein (P04631), a brain injury marker, in the blood of mercury-exposed humans [6]. In the present study, we also found that the Amyloid-β precursor protein (β-APP, P08592), a β-amyloid peptide precursor, was uniquely expressed in the MeHg-exposed group. When deposited after proteolysis, it can form senile plaques, which are characteristic of AD [50]. However, we did not observe modulation of any β-APP proteolytic components. The increase in β-APP accumulation in axons forms endbulbs that cause damage to neuronal communication due to axonal trafficking compromise [23,51], which may affect cerebellar cellular communication between molecular, Purkinje, and granular layers through mossy and climbing fibers.

Through histological analyses, we observed a global neurodegenerative pattern in cerebellar tissue after MeHg exposure due to reductions in Purkinje and granule cell densities. The former receive excitatory outputs from the latter and are essential for precise motor control, coordination, and motor learning [52,53]. In addition, glial cells maintain neural microenvironment homeostasis, playing roles in the immune response, neurochemical balance, and blood–brain barrier (BBB) constitution [31]. The first glial cell population we investigated was the GFAP+ population, which corresponds to astrocytes and Bergman cells in the cerebellum. The findings showed reductions in GFAP immunostained area fractions, these being in accordance with GFAP (P47819) downregulation, as observed via the proteomic approach, which is suggestive of cell density decrease. Underlying the neurodegeneration observed, the increased numbers of microglia modulate the morphological impairment due to their high plasticity [54,55,56]. 

The other two essential components for maintaining neural functions are adequate axon myelinization and synaptic dynamic homeostasis. The myelin sheath is composed of lipidic and protein components, such as cholesterol, sphingomyelin, myelin essential protein (MBP), myelin oligodendrocyte glycoprotein (MOG), and myelin-associated glycoprotein (MAG) [57,58]. The morphological assessment showed decreased immunostained area fractions of MBP in the cerebellums of rats exposed to MeHg, and the proteomic approach showed downregulation of MOG (Q63345) and MAG (P07722), suggesting possible morphological damages to myelin sheaths. In addition, Myelin proteolipid protein (MPP, P60203) regulation was 4.48-fold higher in the MeHg-exposed group. MPP is the most abundant myelin protein in the CNS [57], but, curiously, it was not modulated in rat cerebellums after inorganic mercury intoxication [43], indicating a differential pattern between inorganic and organic mercury. Moreover, in previous studies, rodents with MPP gene overexpression showed a demyelination process that occurs in Pelizaeus–Merzbacher disease [59,60].

Regarding synaptic communication, synaptophysin is a component of the synaptic platform. The proteomic approach showed upregulation of synaptophysin (P07825), which was confirmed by the immunohistochemical analysis that showed increased immunostained area fractions in the exposed group. While the impairment of myelin sheaths may indicate damage to signal transduction through axons, hence functional compromise, the increase in synaptic vesicle markers suggests higher synaptic activity and excitotoxicity [61,62]. The PPI network showed a cluster of several proteins related to synapses with different statuses of regulation, such as the central Dynamin-1 (P21575; upregulated) involved in presynaptic clathrin-mediated endocytosis and exocytosis of vesicles, which is linked to other proteins associated with synaptic plasticity [63]. Moreover, the Circos plot also showed that most synaptic-related proteins were upregulated, such as Synaptojanin-1 (Q62910), reinforcing the synaptic impairments triggered by long-term MeHg exposure.

It is worth mentioning that our results are in accordance with previous findings from other research groups, for instance, those of Shao et al. [64]. The authors analyzed the effects of acute MeHg intoxication in the *Common marmoset,* a nonhuman primate, and found changes in synaptic components, neurotransmitter regulation, and calcium homeostasis. Furthermore, Kong et al. [7] found that rats chronically exposed to MeHg showed impairments to proteins related to calcium homeostasis, energy metabolism, and synaptic signaling in the somatosensory cortex. As for prenatal MeHg exposure, Wang et al. [65] showed the downregulation of synaptophysin in the hippocampi of offspring rats, for example. In vitro studies of granule neurons from the cerebellums of mice showed that MeHg exposure impaired cofilin phosphorylation balance [66], while the study of Shao et al. [67] compared the different response patterns of astrocytes and cerebellar granule neurons upon MeHg exposure and showed the regulation of the cytoskeleton, mitochondria components, and even Huntington’s disease features based on KEGG pathway enrichment analysis. We are aware of the differences in the experimental models and animal species and the ages of the animals used in the different studies; however, when we summarized the global proteomic profile findings, it was evident that, while MeHg neurotoxic effects present a diverse profile, they are associated with some key biological processes in different brain regions.

Finally, the molecular, biochemical, and morphological changes observed were directly associated with motor dysfunction revealed by open field and rotarod tests. Both approaches indicated that the present exposure model efficiently induced clinical features similar to those observed in humans exposed to the toxicant [5]. The open field test evaluated spontaneous locomotion, in which the total distance traveled represented spontaneous horizontal locomotion and the number of rearings represented vertical movement. Although this assay mainly assessed planning and exploratory abilities, both parameters evaluated in this study depend on postural stability, balance, and movement planning. In addition, the increased number of falls through different rotations in the rotarod indicated motor coordination deficits in the animals exposed to MeHg. These findings show that motor damage triggered by MeHg affects cortical and cerebellar functions. In addition to the molecular and biochemical data, such evidence may be useful in designing prevention and diagnosis strategies and studies to investigate the neurological statuses of populations exposed to MeHg over long time periods.

## 5. Conclusions

Our novel results provide essential evidence regarding MeHg neurotoxicity underlying motor damage outcomes. With the mapping of global proteomic profiles, we suggest that, in addition to the classic mechanisms of damage triggered by MeHg, the modulation of synaptic plasticity and myelin integrity plays an essential role in the loss of motor function. Finally, from a translational perspective, the modulated proteins involved in the UPS and HSP systems can also be considered strong candidates for developing neurological damage biomarkers or predictors, in addition to drawing attention to possible associations with already known pathologies. Our study raises new questions about the association of MeHg with neurodegenerative diseases and reinforces the present experimental model as showing promise for pre-clinical studies. There remains a long path to follow to achieve the development of a biomarker with adequate precision, sensitivity, and specificity, especially considering the types of samples and interspecific differential patterns of protein expression involved.

## Data Availability

Not applicable.

## Supplementary Materials

The following supporting information can be downloaded at: https://www.mdpi.com/article/10.3390/toxics10090531/s1, Supplementary Table S1: Identified proteins with expression levels significantly altered in the cerebellums of rats of group exposed to methylmercury (MeHg) vs. the control (C) group.
