# Supplementary material for: Deciphering the Global Proteomic Profile Involved in Methylmercury-Induced Cerebellar Neurodegeneration and Motor Dysfunction in Adult Rats
Source: Toxics. 2022 Sep 9;10(9):531. doi: 10.3390/toxics10090531 (PMC9500842; doi:10.3390/toxics10090531)
Supplement: Supplementary file 1 [file toxics-10-00531-s001.zip › toxics-1892892-supplementary.pdf]

Supplementary Table S1. Identified proteins with expression significantly altered in the cerebellum of rats of group exposed to methylmercury (MeHg) vs. Control (C) group.

Accession	Description	Score	Fold Change
**P60203**	Myelin proteolipid protein	3177	4.48
**Q64541**	Sodium/potassium-transporting ATPase subunit alpha-4	219	4.39
**P06686**	Sodium/potassium-transporting ATPase subunit alpha-2	382	4.35
**P06687**	Sodium/potassium-transporting ATPase subunit alpha-3	431	4.31
**P12847**	Myosin-3	52	3.03
**P07825**	Synaptophysin	326	2.94
**P07340**	Sodium/potassium-transporting ATPase subunit beta-1	2809	2.66
**Q63803**	Guanine nucleotide-binding protein G(s) subunit alpha isoforms XLas	267	2.29
**P63095**	Guanine nucleotide-binding protein G(s) subunit alpha isoforms short	267	2.25
**P38406**	Guanine nucleotide-binding protein G(olf) subunit alpha	254	2.23
**P29348**	Guanine nucleotide-binding protein G(t) subunit alpha-3	254	2.23
**Q6Q7Y5**	Guanine nucleotide-binding protein subunit alpha-13	260	2.23
**P10824**	Guanine nucleotide-binding protein G(i) subunit alpha-1	254	2.18
**P10536**	Ras-related protein Rab-1B	1030	2.16
**P05714**	Ras-related protein Rab-4A	1030	2.16
**P51146**	Ras-related protein Rab-4B	1030	2.16
**P08753**	Guanine nucleotide-binding protein G(k) subunit alpha	254	2.14
**P35284**	Ras-related protein Rab-12	1030	2.14
**P51156**	Ras-related protein Rab-26	1030	2.14
**Q9WVB1**	Ras-related protein Rab-6A	956	2.14
**P04897**	Guanine nucleotide-binding protein G(i) subunit alpha-2	254	2.12
**Q63210**	Guanine nucleotide-binding protein subunit alpha-12	290	2.10
**P61107**	Ras-related protein Rab-14	1096	2.05

**Q05962**	ADP/ATP translocase 1	1756	2.03
**Q09073**	ADP/ATP translocase 2	1565	2.03
**P59215**	Guanine nucleotide-binding protein G(o) subunit alpha	1308	2.01
**P13233**	2'_3'-cyclic-nucleotide 3'-phosphodiesterase	6549	1.99
**P10888**	Cytochrome c oxidase subunit 4 isoform 1_ mitochondrial	792	1.97
**P97546**	Neuroplastin	100	1.93
**Q9JHY2**	Sideroflexin-3	133	1.92
**Q6P6V0**	Glucose-6-phosphate isomerase	855	1.90
**Q9Z270**	Vesicle-associated membrane protein-associated protein A	199	1.84
**P19511**	ATP synthase F(0) complex subunit B1_ mitochondrial	194	1.80
**Q62910**	Synaptojanin-1	76	1.77
**Q9QYU2**	Elongation factor Ts_ mitochondrial	150	1.70
**P11442**	Clathrin heavy chain 1	1098	1.68
**Q63198**	Contactin-1	131	1.67
**P47861**	Synaptotagmin-5	66	1.65
**Q05683**	Glutamate decarboxylase 2	129	1.62
**P35171**	Cytochrome c oxidase subunit 7A2_ mitochondrial	496	1.58
**P11506**	Plasma membrane calcium-transporting ATPase 2	114	1.49
**Q1WIM3**	Cell adhesion molecule 3	506	1.48
**Q641Y2**	NADH dehydrogenase [ubiquinone] iron-sulfur protein 2_ mitochondrial	73	1.48
**Q8VHF5**	Citrate synthase_ mitochondrial	2516	1.46
**O35567**	Bifunctional purine biosynthesis protein PURH	84	1.46
**Q5PPN4**	Carbonic anhydrase-related protein	1752	1.42
**P00406**	Cytochrome c oxidase subunit 2	319	1.40
**P11275**	Calcium/calmodulin-dependent protein kinase type II subunit alpha	521	1.39
**P08413**	Calcium/calmodulin-dependent protein kinase type II subunit beta	1208	1.39
**P62804**	Histone H4	1347<br>7	1.39

**Q64568**	Plasma membrane calcium-transporting ATPase 3	93	1.39
**O35921**	Excitatory amino acid transporter 4	115	1.38
**Q99MZ8**	LIM and SH3 domain protein 1	256	1.38
**P61765**	Syntaxin-binding protein 1	8800	1.38
**P82471**	Guanine nucleotide-binding protein G(q) subunit alpha	124	1.38
**P29995**	Inositol 1_4_5-trisphosphate receptor type 2	76	1.38
**Q5U206**	Calmodulin-like protein 3	1390	1.35
**P25286**	V-type proton ATPase 116 kDa subunit a isoform 1	78	1.35
**Q02253**	Methylmalonate-semialdehyde dehydrogenase [acylating]_ mitochondrial	62	1.35
**Q568Z9**	Phytanoyl-CoA hydroxylase-interacting protein	564	1.35
**P31596**	Excitatory amino acid transporter 2	160	1.34
**P05708**	Hexokinase-1	757	1.34
**P16036**	Phosphate carrier protein_ mitochondrial	642	1.34
**P63329**	Serine/threonine-protein phosphatase 2B catalytic subunit alpha isoform	850	1.34
**Q9QWN8**	Spectrin beta chain_ non-erythrocytic 2	99	1.34
**Q63413**	Spliceosome RNA helicase Ddx39b	448	1.32
**Q64548**	Reticulon-1	204	1.31
**P60881**	Synaptosomal-associated protein 25	305	1.31
**P05197**	Elongation factor 2	172	1.31
**P15791**	Calcium/calmodulin-dependent protein kinase type II subunit delta	572	1.30
**P62898**	Cytochrome c_ somatic	865	1.30
**P06685**	Sodium/potassium-transporting ATPase subunit alpha-1	328	1.30
**P63319**	Protein kinase C gamma type	194	1.28
**P61265**	Syntaxin-1B	456	1.27
**P11730**	Calcium/calmodulin-dependent protein kinase type II subunit gamma	606	1.26
**P54708**	Potassium-transporting ATPase alpha chain 2	238	1.26
**P62716**	Serine/threonine-protein phosphatase 2A catalytic subunit beta isoform	137	1.26
**P63331**	Serine/threonine-protein phosphatase 2A catalytic subunit alpha isoform	137	1.26

**P09626**	Potassium-transporting ATPase alpha chain 1	89	1.25
**P20651**	Serine/threonine-protein phosphatase 2B catalytic subunit beta isoform	173	1.25
**Q9QUL6**	Vesicle-fusing ATPase	929	1.25
**B2RYG6**	Ubiquitin thioesterase OTUB1	208	1.25
**P35571**	Glycerol-3-phosphate dehydrogenase_ mitochondrial	94	1.25
**Q5XI78**	2-oxoglutarate dehydrogenase_ mitochondrial	462	1.23
**Q6PDU7**	ATP synthase subunit g_ mitochondrial	1006	1.23
**Q9JI66**	Electrogenic sodium bicarbonate cotransporter 1	118	1.23
**P97685**	Neurofascin	143	1.23
**P48037**	Annexin A6	125	1.23
**P05696**	Protein kinase C alpha type	64	1.23
**P48675**	Desmin	219	1.22
**P84245**	Histone H3.3	1855	1.22
**Q5BK63**	NADH dehydrogenase [ubiquinone] 1 alpha subcomplex subunit 9_ mitochondrial	229	1.22
**P29101**	Synaptotagmin-2	439	1.22
**Q5XIM9**	T-complex protein 1 subunit beta	144	1.22
**Q9Z0V5**	Peroxiredoxin-4	256	1.22
**P55068**	Brevican core protein	314	1.21
**Q641Z6**	EH domain-containing protein 1	82	1.21
**P12785**	Fatty acid synthase	97	1.21
**P63086**	Mitogen-activated protein kinase 1	104	1.21
**Q9R1Z0**	Voltage-dependent anion-selective channel protein 3	1638	1.21
**P50554**	4-aminobutyrate aminotransferase_ mitochondrial	1432	1.20
**P47860**	ATP-dependent 6-phosphofructokinase_ platelet type	267	1.20
**P04906**	Glutathione S-transferase P	1850	1.19
**P29994**	Inositol 1_4_5-trisphosphate receptor type 1	198	1.19
**Q920L2**	Succinate dehydrogenase [ubiquinone] flavoprotein subunit_ mitochondrial	392	1.19
**P21708**	Mitogen-activated protein kinase 3	165	1.19

**O35095**	Neurochondrin	225	1.19
**Q68FY0**	Cytochrome b-c1 complex subunit 1_ mitochondrial	977	1.17
**Q00729**	Histone H2B type 1-A	1450	1.17
**P04642**	L-lactate dehydrogenase A chain	2181	1.17
**Q63716**	Peroxiredoxin-1	1232	1.17
**Q4V7C7**	Actin-related protein 3	686	1.16
**Q02356**	AMP deaminase 2	78	1.16
**P25809**	Creatine kinase U-type_ mitochondrial	1484	1.16
**O35353**	Guanine nucleotide-binding protein subunit beta-4	2021	1.16
**Q00715**	Histone H2B type 1	9089	1.16
**P13596**	Neural cell adhesion molecule 1	249	1.16
**P81155**	Voltage-dependent anion-selective channel protein 2	2072	1.16
**P62963**	Profilin-1	937	1.16
**P00507**	Aspartate aminotransferase_ mitochondrial	6480	1.15
**P13264**	Glutaminase kidney isoform_ mitochondrial	222	1.15
**P09812**	Glycogen phosphorylase_ muscle form	128	1.15
**Q5RJQ4**	NAD-dependent protein deacetylase sirtuin-2	402	1.15
**P0CG51**	Polyubiquitin-B	9598	1.15
**P50399**	Rab GDP dissociation inhibitor beta	3485	1.15
**P50137**	Transketolase	819	1.15
**Q6P9T8**	Tubulin beta-4B chain	3103<br>1	1.15
**P69897**	Tubulin beta-5 chain	2835<br>1	1.15
**P62982**	Ubiquitin-40S ribosomal protein S27a	9598	1.15
**P62986**	Ubiquitin-60S ribosomal protein L40	9598	1.15
**P63041**	Complexin-1	425	1.15

**P60711**	Actin_ cytoplasmic 1	27341	1.14
**P63259**	Actin_ cytoplasmic 2	27341	1.14
**P63269**	Actin_ gamma-enteric smooth muscle	10743	1.14
**B0K020**	CDGSH iron-sulfur domain-containing protein 1	984	1.14
**P24942**	Excitatory amino acid transporter 1	275	1.14
**Q561S0**	NADH dehydrogenase [ubiquinone] 1 alpha subcomplex subunit 10_ mitochondrial	795	1.14
**P16884**	Neurofilament heavy polypeptide	638	1.14
**P50398**	Rab GDP dissociation inhibitor alpha	5740	1.14
**P63012**	Ras-related protein Rab-3A	1233	1.14
**Q68FR8**	Tubulin alpha-3 chain	13239	1.14
**Q4QRB4**	Tubulin beta-3 chain	20142	1.14
**Q5RKI0**	WD repeat-containing protein 1	206	1.14
**Q9ER34**	Aconitate hydratase_ mitochondrial	3872	1.13
**P68035**	Actin_ alpha cardiac muscle 1	11819	1.13
**P68136**	Actin_ alpha skeletal muscle	11864	1.13
**P62738**	Actin_ aortic smooth muscle	10807	1.13
**P62944**	AP-2 complex subunit beta	286	1.13
**P47858**	ATP-dependent 6-phosphofructokinase_ muscle type	552	1.13
**P09117**	Fructose-bisphosphate aldolase C	11808	1.13

**P09606**	Glutamine synthetase	4449	1.13
**Q63942**	GTP-binding protein Rab-3D	1030	1.13
**P54311**	Guanine nucleotide-binding protein G(I)/G(S)/G(T) subunit beta-1	3116	1.13
**P54313**	Guanine nucleotide-binding protein G(I)/G(S)/G(T) subunit beta-2	1913	1.13
**Q6LED0**	Histone H3.1	380	1.13
**P42123**	L-lactate dehydrogenase B chain	9037	1.13
**P04631**	Protein S100-B	17011	1.13
**P63055**	Purkinje cell protein 4	1493	1.13
**Q63941**	Ras-related protein Rab-3B	1030	1.13
**Q53B90**	Ras-related protein Rab-43	1030	1.13
**P13638**	Sodium/potassium-transporting ATPase subunit beta-2	317	1.13
**P68370**	Tubulin alpha-1A chain	17344	1.13
**Q6P9V9**	Tubulin alpha-1B chain	19019	1.13
**Q5U300**	Ubiquitin-like modifier-activating enzyme 1	313	1.13
**P10818**	Cytochrome c oxidase subunit 6A1_ mitochondrial	2261	1.12
**P05065**	Fructose-bisphosphate aldolase A	5704	1.12
**P52287**	Guanine nucleotide-binding protein G(I)/G(S)/G(T) subunit beta-3	719	1.12
**O88989**	Malate dehydrogenase_ cytoplasmic	6766	1.12
**Q66HF1**	NADH-ubiquinone oxidoreductase 75 kDa subunit_ mitochondrial	512	1.12
**P35281**	Ras-related protein Rab-10	1030	1.12
**Q5U316**	Ras-related protein Rab-35	1088	1.12
**P62824**	Ras-related protein Rab-3C	1030	1.12
**P07632**	Superoxide dismutase [Cu-Zn]	2891	1.12
**P21707**	Synaptotagmin-1	231	1.12

**P48500**	Triosephosphate isomerase	11764	1.12
**Q6AYZ1**	Tubulin alpha-1C chain	13257	1.12
**Q5XIF6**	Tubulin alpha-4A chain	17103	1.12
**Q6AY56**	Tubulin alpha-8 chain	7864	1.12
**P85108**	Tubulin beta-2A chain	27779	1.12
**Q3KRE8**	Tubulin beta-2B chain	26903	1.12
**Q66HA8**	Heat shock protein 105 kDa	76	1.12
**P35289**	Ras-related protein Rab-15	957	1.12
**Q6NYB7**	Ras-related protein Rab-1A	1030	1.12
**P52303**	AP-1 complex subunit beta-1	139	1.12
**P45592**	Cofilin-1	6763	1.11
**P11240**	Cytochrome c oxidase subunit 5A_ mitochondrial	3967	1.11
**Q6P6R2**	Dihydrolipoyl dehydrogenase_ mitochondrial	1018	1.11
**P04636**	Malate dehydrogenase_ mitochondrial	15278	1.11
**P54921**	Alpha-soluble NSF attachment protein	482	1.11
**P11030**	Acyl-CoA-binding protein	741	1.09
**P31399**	ATP synthase subunit d_ mitochondrial	3831	1.09
**P30904**	Macrophage migration inhibitory factor	4630	1.09
**P11980**	Pyruvate kinase PKM	10257	1.09
**B0BNF1**	Septin-8	486	1.09
**P35280**	Ras-related protein Rab-8A	1030	1.09

**P70550**	Ras-related protein Rab-8B	1030	1.09
**P13221**	Aspartate aminotransferase_ cytoplasmic	5532	1.08
**P62161**	Calmodulin	10414	1.08
**P20788**	Cytochrome b-c1 complex subunit Rieske_ mitochondrial	1645	1.08
**P14408**	Fumarate hydratase_ mitochondrial	541	1.08
**Q5XHZ0**	Heat shock protein 75 kDa_ mitochondrial	1871	1.08
**P25113**	Phosphoglycerate mutase 1	9132	1.08
**P52873**	Pyruvate carboxylase_ mitochondrial	92	1.08
**P12928**	Pyruvate kinase PKLR	939	1.08
**Q05140**	Clathrin coat assembly protein AP180	129	1.08
**P19944**	60S acidic ribosomal protein P1	1399	1.08
**P84087**	Complexin-2	2305	1.08
**Q78P75**	Dynein light chain 2_ cytoplasmic	777	1.08
**Q5M964**	Fumarate hydratase 1	541	1.07
**Q9ESV6**	Glyceraldehyde-3-phosphate dehydrogenase_ testis-specific	1881	1.07
**P02262**	Histone H2A type 1	24276	1.07
**P0C169**	Histone H2A type 1-C	24276	1.07
**P0C170**	Histone H2A type 1-E	24276	1.07
**Q64598**	Histone H2A type 1-F	24276	1.07
**P0CC09**	Histone H2A type 2-A	24276	1.07
**Q4FZT6**	Histone H2A type 3	24276	1.07

**Q00728**	Histone H2A type 4	24276	1.07
**A9UMV8**	Histone H2A.J	24276	1.07
**P10111**	Peptidyl-prolyl cis-trans isomerase A	5940	1.07
**P26284**	Pyruvate dehydrogenase E1 component subunit alpha_ somatic form_ mitochondrial	902	1.07
**P16086**	Spectrin alpha chain_ non-erythrocytic 1	906	1.07
**P09811**	Glycogen phosphorylase_ liver form	158	1.07
**P23565**	Alpha-internexin	2848	1.06
**P04797**	Glyceraldehyde-3-phosphate dehydrogenase	23558	1.06
**P19527**	Neurofilament light polypeptide	4078	1.06
**P49432**	Pyruvate dehydrogenase E1 component subunit beta_ mitochondrial	2178	1.06
**Q63537**	Synapsin-2	1909	1.06
**Q9Z2L0**	Voltage-dependent anion-selective channel protein 1	4505	1.06
**P07171**	Calbindin	5179	1.05
**Q99NA5**	Isocitrate dehydrogenase [NAD] subunit alpha_ mitochondrial	1461	1.05
**P31044**	Phosphatidylethanolamine-binding protein 1	15008	1.05
**O88767**	Protein DJ-1	6592	1.05
**Q8VBU2**	Protein NDRG2	1790	1.05
**P11348**	Dihydropteridine reductase	1097	1.05
**Q63028**	Alpha-adducin	306	1.05
**P47942**	Dihydropyrimidinase-related protein 2	13177	1.04
**P10860**	Glutamate dehydrogenase 1_ mitochondrial	1391	1.04
**Q6IMY8**	Heterogeneous nuclear ribonucleoprotein U	454	1.04
**P21575**	Dynamin-1	2040	1.03

**P62630**	Elongation factor 1-alpha 1	4142	1.03
**P62815**	V-type proton ATPase subunit B_ brain isoform	889	1.03
**P08461**	Dihydrolipoyllysine-residue acetyltransferase component of pyruvate dehydrogenase complex_ mitochondrial	1006	1.02
**P14659**	Heat shock-related 70 kDa protein 2	2013	1.02
**P82995**	Heat shock protein HSP 90-alpha	3377	1.02
**P20761**	Ig gamma-2B chain C region	113	-0.44
**B0BNE5**	S-formylglutathione hydrolase	200	-0.46
**P02680**	Fibrinogen gamma chain	118	-0.49
**P18421**	Proteasome subunit beta type-1	128	-0.52
**P63170**	Dynein light chain 1_ cytoplasmic	377	-0.65
**P11517**	Hemoglobin subunit beta-2	1177<br>4	-0.65
**Q62936**	Disks large homolog 3	361	-0.68
**P01946**	Hemoglobin subunit alpha-1/2	4748<br>5	-0.69
**Q6Q0N1**	Cytosolic non-specific dipeptidase	266	-0.70
**P07722**	Myelin-associated glycoprotein	158	-0.73
**Q68A21**	Transcriptional activator protein Pur-beta	285	-0.73
**P02091**	Hemoglobin subunit beta-1	1795<br>9	-0.73
**Q99MZ4**	Gamma-glutamyltransferase 7	98	-0.75
**P97686**	Neuronal cell adhesion molecule	62	-0.76
**P15146**	Microtubule-associated protein 2	387	-0.76
**P13803**	Electron transfer flavoprotein subunit alpha_ mitochondrial	287	-0.78
**P70615**	Lamin-B1	150	-0.79
**P47875**	Cysteine and glycine-rich protein 1	110	-0.79
**P08081**	Clathrin light chain A	360	-0.80

**B0BNN3**	Carbonic anhydrase 1	441	-0.81
**Q5XIH7**	Prohibitin-2	362	-0.81
**Q63560**	Microtubule-associated protein 6	212	-0.82
**Q9Z0W5**	Protein kinase C and casein kinase substrate in neurons protein 1	262	-0.82
**P22062**	Protein-L-isoaspartate(D-aspartate) O-methyltransferase	2289	-0.82
**P31000**	Vimentin	1278	-0.82
**O08838**	Amphiphysin	251	-0.83
**P39069**	Adenylate kinase isoenzyme 1	2086	-0.84
**Q63345**	Myelin-oligodendrocyte glycoprotein	446	-0.84
**P35435**	ATP synthase subunit gamma_ mitochondrial	490	-0.85
**P13084**	Nucleophosmin	162	-0.86
**Q9JHU0**	Dihydropyrimidinase-related protein 5	185	-0.86
**Q8K586**	GTP-binding nuclear protein Ran_ testis-specific isoform	714	-0.86
**Q5U318**	Astrocytic phosphoprotein PEA-15	376	-0.87
**P11884**	Aldehyde dehydrogenase_ mitochondrial	242	-0.88
**Q9Z0V6**	Thioredoxin-dependent peroxide reductase_ mitochondrial	1026	-0.88
**P15205**	Microtubule-associated protein 1B	103	-0.90
**P37377**	Alpha-synuclein	903	-0.90
**P48721**	Stress-70 protein_ mitochondrial	369	-0.90
**Q00981**	Ubiquitin carboxyl-terminal hydrolase isozyme L1	1593	-0.90
**P35434**	ATP synthase subunit delta_ mitochondrial	360	-0.90
**P19332**	Microtubule-associated protein tau	544	-0.90
**P02625**	Parvalbumin alpha	9319	-0.90
**P09951**	Synapsin-1	2155	-0.90
**P18418**	Calreticulin	759	-0.91
**P47819**	Glial fibrillary acidic protein	7331	-0.91
**Q7TPB1**	T-complex protein 1 subunit delta	434	-0.92
**P11598**	Protein disulfide-isomerase A3	382	-0.92

**Q6URK4**	Heterogeneous nuclear ribonucleoprotein A3	1085	-0.92
**P62959**	Histidine triad nucleotide-binding protein 1	2216	-0.92
**P20760**	Ig gamma-2A chain C region	1035	-0.92
**P34926**	Microtubule-associated protein 1A	189	-0.92
**F1LRL9**	Microtubule-associated protein 1B	107	-0.92
**P19804**	Nucleoside diphosphate kinase B	3358	-0.92
**O35964**	Endophilin-A2	195	-0.93
**P84082**	ADP-ribosylation factor 2	658	-0.93
**A7VJC2**	Heterogeneous nuclear ribonucleoproteins A2/B1	2659	-0.93
**O08839**	Myc box-dependent-interacting protein 1	1103	-0.94
**P19234**	NADH dehydrogenase [ubiquinone] flavoprotein 2_ mitochondrial	1218	-0.94
**P26772**	10 kDa heat shock protein_ mitochondrial	2966	-0.95
**P35213**	14-3-3 protein beta/alpha	4945	-0.95
**P68511**	14-3-3 protein eta	5121	-0.95
**Q05982**	Nucleoside diphosphate kinase A	1512	-0.95
**Q9JJ54**	Heterogeneous nuclear ribonucleoprotein D0	479	-0.96
**O35179**	Endophilin-A1	1195	-0.96
**P39052**	Dynamin-2	435	-0.97
**P63039**	60 kDa heat shock protein_ mitochondrial	1275	-0.97
**P61980**	Heterogeneous nuclear ribonucleoprotein K	2471	-0.97
**P62260**	14-3-3 protein epsilon	6626	-0.97
**Q63754**	Beta-synuclein	6337	-0.97
**P61983**	14-3-3 protein gamma	6504	-0.98
**P63102**	14-3-3 protein zeta/delta	10182	-0.98
**P10719**	ATP synthase subunit beta_ mitochondrial	18120	-0.98
**Q5XIB3**	Inactive ADP-ribosyltransferase ARH2	122	Control

**Q64536**	[Pyruvate dehydrogenase (acetyl-transferring)] kinase isozyme 2, mitochondrial	316	Control
**O88484**	[Pyruvate dehydrogenase [acetyl-transferring]]-phosphatase 2, mitochondrial	70	Control
**Q63569**	26S protease regulatory subunit 6A	77	Control
**P02401**	60S acidic ribosomal protein P2	721	Control
**Q6AYP7**	7-methylguanosine phosphate-specific 5'-nucleotidase	90	Control
**P49911**	Acidic leucine-rich nuclear phosphoprotein 32 family member A	192	Control
**Q99PD4**	Actin-related protein 2/3 complex subunit 1A	71	Control
**O88656**	Actin-related protein 2/3 complex subunit 1B	121	Control
**P85970**	Actin-related protein 2/3 complex subunit 2	182	Control
**Q6SKG1**	Acyl-coenzyme A synthetase ACSM3_ mitochondrial	94	Control
**Q9QYL8**	Acyl-protein thioesterase 2	188	Control
**Q9WUS0**	Adenylate kinase 4_ mitochondrial	320	Control
**Q68FP8**	Adenylate kinase 8	86	Control
**O88923**	Adhesion G protein-coupled receptor L2	72	Control
**Q8CG45**	Aflatoxin B1 aldehyde reductase member 2	197	Control
**Q5QD51**	A-kinase anchor protein 12	102	Control
**P0C6C0**	A-kinase anchor protein SPHKAP	74	Control
**P50475**	Alanine--tRNA ligase_ cytoplasmic	122	Control
**Q64563**	Alcohol dehydrogenase 4	77	Control
**Q66HG4**	Aldose 1-epimerase	119	Control
**P07943**	Aldose reductase	124	Control
**P21396**	Amine oxidase [flavin-containing] A	80	Control
**Q6AYS7**	Aminoacylase-1A	84	Control
**Q6PTT0**	Aminoacylase-1B	101	Control
**Q07936**	Annexin A2	165	Control
**P14668**	Annexin A5	158	Control
**Q32Q06**	AP-1 complex subunit mu-1	179	Control
**Q32KJ9**	Arylsulfatase G	83	Control

**P29419**	ATP synthase subunit e_ mitochondrial	395	Control
**O70595**	ATP-binding cassette sub-family B member 6_ mitochondrial	104	Control
**Q5U216**	ATP-dependent RNA helicase DDX39A	263	Control
**Q9R1T1**	Barrier-to-autointegration factor	337	Control
**Q641X3**	Beta-hexosaminidase subunit alpha	90	Control
**P07882**	Bile salt-activated lipase	77	Control
**Q5BK10**	Calpain-13	58	Control
**P09456**	cAMP-dependent protein kinase type I-alpha regulatory subunit	100	Control
**Q63704**	Carnitine O-palmitoyltransferase 1_ muscle isoform	66	Control
**P19139**	Casein kinase II subunit alpha	126	Control
**Q1M168**	Caytaxin	579	Control
**Q5M7A7**	CB1 cannabinoid receptor-interacting protein 1	1300	Control
**O35112**	CD166 antigen	102	Control
**Q01134**	Choline kinase alpha	87	Control
**P08082**	Clathrin light chain B	317	Control
**Q99M76**	Cohesin subunit SA-3	85	Control
**Q9Z1T4**	Connector enhancer of kinase suppressor of ras 2	54	Control
**H1UBN0**	Copine-7	128	Control
**Q5BJS7**	Copine-9	60	Control
**Q9Z2F5**	C-terminal-binding protein 1	192	Control
**Q99P39**	Cysteine desulfurase_ mitochondrial	74	Control
**P10715**	Cytochrome c_ testis-specific	185	Control
**Q9QXU8**	Cytoplasmic dynein 1 light intermediate chain 1	89	Control
**O35078**	D-amino-acid oxidase	200	Control
**P36365**	Dimethylaniline monooxygenase [N-oxide-forming] 1	79	Control
**Q01460**	Di-N-acetylchitobiase	78	Control
**O55096**	Dipeptidyl peptidase 3	81	Control
**O54747**	DNA polymerase delta catalytic subunit	65	Control

**D3ZCM9**	Dynein assembly factor 3_ axonemal	47	Control
**Q68FR6**	Elongation factor 1-gamma	123	Control
**Q8K3M6**	ERC protein 2	69	Control
**B5DEH2**	Erlin-2	126	Control
**Q5RJL0**	Ermin	129	Control
**P85845**	Fascin	73	Control
**P02793**	Ferritin light chain 1	150	Control
**O55145**	Fractalkine	86	Control
**B2RYW9**	Fumarylacetoacetate hydrolase domain-containing protein 2	211	Control
**P38552**	Galectin-4	98	Control
**P04634**	Gastric triacylglycerol lipase	97	Control
**Q9Z339**	Glutathione S-transferase omega-1	302	Control
**Q0VGK4**	Glycerophosphodiester phosphodiesterase domain-containing protein 1	149	Control
**G3V7G8**	Glycine--tRNA ligase	70	Control
**P35053**	Glypican-1	64	Control
**Q4V7C6**	GMP synthase [glutamine-hydrolyzing]	180	Control
**D3ZZL9**	GRIP and coiled-coil domain-containing protein 2	48	Control
**Q925G1**	Hepatoma-derived growth factor-related protein 2	52	Control
**Q923W4**	Hepatoma-derived growth factor-related protein 3	130	Control
**P62749**	Hippocalcin-like protein 1	332	Control
**P06349**	Histone H1t	101	Control
**Q9Z214**	Homer protein homolog 1	95	Control
**O88801**	Homer protein homolog 2	95	Control
**P50503**	Hsc70-interacting protein	151	Control
**Q63692**	Hsp90 co-chaperone Cdc37	81	Control
**Q63617**	Hypoxia up-regulated protein 1	108	Control
**P20759**	Ig gamma-1 chain C region	150	Control
**F1M5M3**	Inactive serine/threonine-protein kinase TEX14	88	Control

**Q9QYE7**	Integrin alpha-D	63	Control
**P56574**	Isocitrate dehydrogenase [NADP]_ mitochondrial	171	Control
**Q6IMF3**	Keratin_ type II cytoskeletal 1	41	Control
**Q6IG02**	Keratin_ type II cytoskeletal 2 epidermal	66	Control
**Q6IG00**	Keratin_ type II cytoskeletal 4	191	Control
**Q4FZU2**	Keratin_ type II cytoskeletal 6A	125	Control
**Q6IG12**	Keratin_ type II cytoskeletal 7	125	Control
**Q6IG03**	Keratin_ type II cytoskeletal 73	66	Control
**Q6IG05**	Keratin_ type II cytoskeletal 75	191	Control
**Q10758**	Keratin_ type II cytoskeletal 8	125	Control
**Q64602**	Kynurenine/alpha-aminoadipate aminotransferase_ mitochondrial	67	Control
**Q6P7Q4**	Lactoylglutathione lyase	331	Control
**P19629**	L-lactate dehydrogenase C chain	146	Control
**P15650**	Long-chain specific acyl-CoA dehydrogenase_ mitochondrial	91	Control
**Q63341**	Macrophage metalloelastase	81	Control
**Q62667**	Major vault protein	54	Control
**Q5M7W5**	Microtubule-associated protein 4	81	Control
**Q5XIT1**	Microtubule-associated protein RP/EB family member 3	495	Control
**Q62625**	Microtubule-associated proteins 1A/1B light chain 3B	438	Control
**P22199**	Mineralocorticoid receptor	89	Control
**Q4KM98**	Mitochondrial fission factor	156	Control
**Q64119**	Myosin light polypeptide 6	331	Control
**Q64122**	Myosin regulatory light polypeptide 9	81	Control
**Q62812**	Myosin-9	69	Control
**Q769K2**	N-acyl-phosphatidylethanolamine-hydrolyzing phospholipase D	104	Control
**P28841**	Neuroendocrine convertase 2	58	Control
**P12389**	Neuronal acetylcholine receptor subunit alpha-2	179	Control
**P12392**	Neuronal acetylcholine receptor subunit beta-4	118	Control

**Q62718**	Neurotrimin	98	Control
**Q63525**	Nuclear migration protein nudC	51	Control
**Q6AYD9**	Nucleoside diphosphate-linked moiety X motif 19	116	Control
**Q4R180**	Origin recognition complex subunit 3	115	Control
**Q4V8B0**	Oxidation resistance protein 1	145	Control
**Q9QVC8**	Peptidyl-prolyl cis-trans isomerase FKBP4	85	Control
**P53812**	Phosphatidylinositol transfer protein beta isoform	109	Control
**B1WBU8**	Pleckstrin homology domain-containing family D member 1	89	Control
**Q66H20**	Polypyrimidine tract-binding protein 2	77	Control
**P20267**	POU domain_ class 3_ transcription factor 1	86	Control
**P56222**	POU domain_ class 3_ transcription factor 2	86	Control
**Q63262**	POU domain_ class 3_ transcription factor 3	86	Control
**P62516**	POU domain_ class 3_ transcription factor 4	86	Control
**P48679**	Prelamin-A/C	76	Control
**A1A5S1**	Pre-mRNA-processing factor 6	59	Control
**Q80ZG5**	Pre-mRNA-splicing factor SLU7	122	Control
**Q9EPC6**	Profilin-2	318	Control
**P83868**	Prostaglandin E synthase 3	215	Control
**Q5BK81**	Prostaglandin reductase 2	72	Control
**P18420**	Proteasome subunit alpha type-1	212	Control
**Q6MGD0**	Protein CutA	205	Control
**Q63081**	Protein disulfide-isomerase A6	150	Control
**Q9Z250**	Protein lin-7 homolog A	136	Control
**Q6JE36**	Protein NDRG1	205	Control
**P35467**	Protein S100-A1	283	Control
**P47709**	Rabphilin-3A	74	Control
**Q62796**	RalA-binding protein 1	136	Control
**Q5XXR3**	Rho guanine nucleotide exchange factor 6	76	Control

**Q68FQ7**	RNA polymerase II-associated protein 3	71	Control
**P47868**	Secretogranin-3	110	Control
**D4A2H2**	Serine palmitoyltransferase_ long chain base subunit 1	188	Control
**D3ZHP7**	Serine/threonine-protein kinase ULK3	76	Control
**P36876**	Serine/threonine-protein phosphatase 2A 55 kDa regulatory subunit B alpha isoform	96	Control
**Q4PJT6**	Spermatogenesis-associated protein 24	97	Control
**Q6PEC4**	S-phase kinase-associated protein 1	802	Control
**P32851**	Syntaxin-1A	96	Control
**Q66HA4**	Tax1-binding protein 1 homolog	48	Control
**Q5CD77**	TBC1 domain family member 14	116	Control
**Q4V8G8**	Tektin-3	76	Control
**Q9Z311**	Trans-2-enoyl-CoA reductase_ mitochondrial	94	Control
**P26342**	Transforming growth factor beta receptor type 3	71	Control
**Q6AY76**	Transmembrane protein 248	114	Control
**A0A0G2JXN2**	Tripartite motif protein 46 (Predicted)	80	Control
**Q64560**	Tripeptidyl-peptidase 2	79	Control
**Q6PCT3**	Tumor protein D54	122	Control
**Q7M767**	Ubiquitin-conjugating enzyme E2 variant 2	878	Control
**Q4KM73**	UMP-CMP kinase	243	Control
**P62762**	Visinin-like protein 1	386	Control
**Q8VHW7**	Voltage-dependent calcium channel gamma-6 subunit	90	Control
**Q9ERH3**	WD repeat-containing protein 7	60	Control
**P35738**	2-oxoisovalerate dehydrogenase subunit beta_ mitochondrial	102	MeHg
**P62909**	40S ribosomal protein S3	62	MeHg
**O35552**	6-phosphofructo-2-kinase/fructose-2_6-bisphosphatase 3	58	MeHg
**Q8VH46**	Actin filament-associated protein 1	60	MeHg
**P69682**	Adaptin ear-binding coat-associated protein 1	165	MeHg

**P13601**	Aldehyde dehydrogenase_ cytosolic 1	66	MeHg
**P08592**	Amyloid-beta precursor protein	75	MeHg
**Q5U312**	Ankycorbin	55	MeHg
**F1LQ70**	Arachidonate 12-lipoxygenase_ 12S-type	93	MeHg
**P50430**	Arylsulfatase B	101	MeHg
**D3ZAF6**	ATP synthase subunit f_ mitochondrial	683	MeHg
**Q9QY44**	ATP-binding cassette sub-family D member 2	96	MeHg
**Q701R3**	Beta-galactoside alpha-2_6-sialyltransferase 2	58	MeHg
**O88881**	Brain-enriched guanylate kinase-associated protein	74	MeHg
**P24268**	Cathepsin D	274	MeHg
**P23505**	Cell surface glycoprotein gp42	178	MeHg
**P18395**	Cold shock domain-containing protein E1	74	MeHg
**P00564**	Creatine kinase M-type	55	MeHg
**P97536**	Cullin-associated NEDD8-dissociated protein 1	46	MeHg
**Q63270**	Cytoplasmic aconitate hydratase	80	MeHg
**Q68FS4**	Cytosol aminopeptidase	137	MeHg
**Q8CF97**	Deubiquitinating protein VCIP135	67	MeHg
**P55266**	Double-stranded RNA-specific adenosine deaminase	48	MeHg
**P51400**	Double-stranded RNA-specific editase 1	77	MeHg
**B5DF91**	ELAV-like protein 1	79	MeHg
**Q8CH84**	ELAV-like protein 2	104	MeHg
**O09032**	ELAV-like protein 4	104	MeHg
**A0A140TAF2**	ELAV-like protein	104	MeHg
**Q499U2**	Engulfment and cell motility protein 3	158	MeHg
**P54759**	Ephrin type-A receptor 7	48	MeHg
**Q5XI72**	Eukaryotic translation initiation factor 4H	156	MeHg
**Q04931**	FACT complex subunit SSRP1	66	MeHg

**D3ZHA0**	Filamin-C	43	MeHg
**O88387**	FYVE_ RhoGEF and PH domain-containing protein 4	53	MeHg
**O09028**	Gamma-aminobutyric acid receptor subunit pi	89	MeHg
**Q63226**	Glutamate receptor ionotropic_ delta-2	58	MeHg
**P30713**	Glutathione S-transferase theta-2	233	MeHg
**P46413**	Glutathione synthetase	93	MeHg
**Q5I0P2**	Glycine cleavage system H protein_ mitochondrial	358	MeHg
**Q6AYR6**	Haloacid dehalogenase-like hydrolase domain-containing protein 2	92	MeHg
**P27926**	Hexokinase-3	72	MeHg
**Q5XI06**	Histone acetyltransferase KAT8	73	MeHg
**D4A1C2**	Junction adhesion molecule-like	78	MeHg
**Q6IFU7**	Keratin_ type I cytoskeletal 42	117	MeHg
**Q6IG04**	Keratin_ type II cytoskeletal 72	67	MeHg
**Q91V33**	KH domain-containing_ RNA-binding_ signal transduction-associated protein 1	74	MeHg
**F1M4A4**	Kinesin family member 1A	89	MeHg
**Q811X6**	Lambda-crystallin homolog	96	MeHg
**Q5XIN6**	LETM1 and EF-hand domain-containing protein 1_ mitochondrial	92	MeHg
**P30349**	Leukotriene A-4 hydrolase	61	MeHg
**P10867**	L-gulonolactone oxidase	47	MeHg
**D3ZAP3**	Microtubule-associated protein 10	57	MeHg
**Q9Z2A6**	Mitogen-activated protein kinase 15	78	MeHg
**D3ZG83**	Mitogen-activated protein kinase kinase kinase 10	56	MeHg
**O55164**	Multiple PDZ domain protein	35	MeHg
**Q80W89**	NADH dehydrogenase [ubiquinone] 1 alpha subcomplex subunit 11	540	MeHg
**P55161**	Nck-associated protein 1	90	MeHg
**P49791**	Nuclear pore complex protein Nup153	71	MeHg
**Q9ES54**	Nuclear protein localization protein 4 homolog	48	MeHg
**Q8R5M4**	Optineurin	48	MeHg

**Q9JLN4**	Peptidoglycan recognition protein 1	91	MeHg
**Q9Z1L0**	Phosphatidylinositol 4_5-bisphosphate 3-kinase catalytic subunit beta isoform	48	MeHg
**Q6AYN4**	Phytanoyl-CoA hydroxylase-interacting protein-like	164	MeHg
**P11505**	Plasma membrane calcium-transporting ATPase 1	63	MeHg
**P20786**	Platelet-derived growth factor receptor alpha	55	MeHg
**Q05030**	Platelet-derived growth factor receptor beta	67	MeHg
**O88758**	Potassium voltage-gated channel subfamily S member 1	80	MeHg
**D3ZFB6**	Proline-rich transmembrane protein 2	151	MeHg
**P23606**	Protein-glutamine gamma-glutamyltransferase K	87	MeHg
**P70600**	Protein-tyrosine kinase 2-beta	31	MeHg
**Q6AYT0**	Quinone oxidoreductase	93	MeHg
**F1M386**	Rap guanine nucleotide exchange factor 2	29	MeHg
**Q62656**	Receptor-type tyrosine-protein phosphatase zeta	33	MeHg
**O08774**	Regulator of G-protein signaling 12	53	MeHg
**P49803**	Regulator of G-protein signaling 7	48	MeHg
**O55005**	Roundabout homolog 1	156	MeHg
**Q6AY30**	Saccharopine dehydrogenase-like oxidoreductase	86	MeHg
**Q5EB96**	Septin-1	58	MeHg
**Q62726**	Serine/threonine-protein kinase ICK	103	MeHg
**Q9R011**	Serine/threonine-protein kinase PLK3	83	MeHg
**D3ZMK9**	Similar to DNA segment_ Chr 8_ ERATO Doi 82_ expressed (Predicted)_ isoform CRA_a	66	MeHg
**Q63633**	Solute carrier family 12 member 5	70	MeHg
**Q66HR0**	Solute carrier family 12 member 9	56	MeHg
**Q7TNZ6**	STE20-related kinase adapter protein alpha	85	MeHg
**P97690**	Structural maintenance of chromosomes protein 3	45	MeHg
**P21913**	Succinate dehydrogenase [ubiquinone] iron-sulfur subunit_ mitochondrial	128	MeHg
**Q68FU8**	SURP and G-patch domain-containing protein 1	54	MeHg
**Q02563**	Synaptic vesicle glycoprotein 2A	46	MeHg

**P97610**	Synaptotagmin-12	175	MeHg
**Q9Z220**	Testis-specific gene 10 protein	62	MeHg
**Q6AY87**	THO complex subunit 6 homolog	83	MeHg
**Q5XHY5**	Threonine--tRNA ligase_ cytoplasmic	44	MeHg
**Q9EPI8**	Transcription termination factor 1_ mitochondrial	54	MeHg
**Q925B3**	Transient receptor potential cation channel subfamily M member 7	59	MeHg
**Q8K582**	tRNA-dihydrouridine(16/17) synthase [NAD(P)(+)]-like	66	MeHg
**Q6P7B0**	Tryptophan--tRNA ligase_ cytoplasmic	126	MeHg
**Q5U2Y6**	Tuftelin-interacting protein 11	46	MeHg
**Q5RJR2**	Twinfilin-1	99	MeHg
**Q9QWG5**	Type II inositol 3_4-bisphosphate 4-phosphatase	83	MeHg
**P32577**	Tyrosine-protein kinase CSK	70	MeHg
**P41499**	Tyrosine-protein phosphatase non-receptor type 11	48	MeHg
**Q4VSI4**	Ubiquitin carboxyl-terminal hydrolase 7	49	MeHg
**P53767**	Vascular endothelial growth factor receptor 1	79	MeHg
**O08775**	Vascular endothelial growth factor receptor 2	62	MeHg
**Q91ZT1**	Vascular endothelial growth factor receptor 3	63	MeHg
**G3V893**	Zinc finger protein 335	66	MeHg

[a]Accession ID according to Uniport.org database. Positive and negative values of fold change indicate up- and down-regulated proteins, respectively. The identification with MeHg or Control in the fourth column means exclusive expression in the exposed or control group, respectively. Results of the comparison between the MeHg group *versus* the control group.
